# Supplementary material for: Single-cell analysis based dissection of clonality in myelofibrosis
Source: Nat Commun. 2020 Jan 7;11:73. doi: 10.1038/s41467-019-13892-x (PMC6946829; doi:10.1038/s41467-019-13892-x)
Supplement: Supplementary file 1 — Supplementary Information [file 41467_2019_13892_MOESM1_ESM.pdf]

Supplementary Information

Single-cell analysis based dissection of clonality in myelofibrosis

Elena Mylonas[1*], Kenichi Yoshida[2*], Mareike Frick[1*], Kaja Hoyer[1], Friederike Christen[1], Jaspal Kaeda[1], Matthias Obenaus[1], Daniel Noerenberg[1], Cornelius Hennch[1], Willy Chan[1], Yotaro Ochi[2,3], Yuichi Shiraishi[4], Yusuke Shiozawa[2], Thorsten Zenz[5], Christopher C. Oakes[6], Birgit Sawitzki[7], Michaela Schwarz[1], Lars Bullinger[1,10], Philipp le Coutre[1], Matthew J.J Rose-Zerilli[8], Seishi Ogawa[2,3,9], and Frederik Damm[1,10]

1. Charité – Universitätsmedizin Berlin, corporate member of Freie Universität Berlin, Humboldt-Universität zu Berlin, and Berlin Institute of Health, Department of Hematology, Oncology, and Tumor Immunology, Berlin, Germany
2. Department of Pathology and Tumor Biology, Graduate School of Medicine, Kyoto University, Kyoto, Japan
3. Institute for the Advanced Study of Human Biology (WPI-ASHBi), Kyoto University, Kyoto, Japan
4. Laboratory of Sequence Analysis, Human Genome Center, Institute of Medical Science, The University of Tokyo, Tokyo, Japan
5. Department of Medical Oncology and Hematology, University Hospital Zurich / University of Zurich, Zurich, Switzerland
6. Division of Hematology, Department of Internal Medicine, The Ohio State University, Columbus, OH, USA
7. Charité – Universitätsmedizin Berlin, corporate member of Freie Universität Berlin, Humboldt-Universität zu Berlin, and Berlin Institute of Health, Institute for Medical Immunology, Berlin, Germany
8. Cancer Sciences, Faculty of Medicine, University of Southampton, Southampton, United Kingdom
9. Department of Medicine, Centre for Haematology and Regenerative Medicine, Karolinska Institute, Stockholm, Sweden
10. German Cancer Consortium (DKTK) and German Cancer Research Center (DKFZ), Heidelberg, Germany

* These authors contributed equally to this work.

Supplementary Tables

Supplementary Table 1: Antibodies and their respective dilutions used for flow-cytometry and cell-sorting experiments.

Name	Conjugate	Dilution	Company	Catalogue Number	Clone
CD34-PE	PE	1:5	BD	555822	581
Streptavidine-BV	BV421	1:500	Biolegend	405225	
CD66b-PE	PE	1:60	BD	561650	G10F5
CD3-FITC	FITC	1:40	BD	555339	HIT3a
CD19-PECy7	PE-Cy7	1:100	BD	560728	HIB19
CD14-APC	APC	1:20	eBioscience	17-0149	61D3

Supplementary Table 2: Quality control assessment of single-cell flow-sorting by parallel plate processing of two copy number probes (*SLC2A9* and *PPIP5K1* located in diploid regions of the genome) by qPCR.

	PPIPK5		*SCL2A9*		Doublets rate		Empty wells rate	
**Patient**	**Doublet rate**	**Empty well**	**Doublet rate**	**Empty well**	**Mean**	**STD.Dev**	**Mean**	**STD.Dev**
MPN01_t1	4.76	8.35	3.17	6.35	3.97	1.12	7.35	1.41
MPN01_t2	3.17	4.76	7.94	4.76	5.56	3.37	4.76	0.00
MPN04_t1	0	7.94	3.17	11.11	1.59	2.24	9.53	2.24
MPN04_t2	1.59	14.29	1.72	15.58	1.66	0.09	14.94	0.91
MPN05	9.68	11.29	3.23	11.29	6.46	4.56	11.29	0.00
MPN10_t1	3.17	4.76	3.17	7.94	3.17	0.00	6.35	2.25
MPN10_t2	10	10	4.92	9.84	7.46	3.59	9.92	0.11
MPN11_t2	0	12.9	0	11.11	0.00	0.00	12.01	1.27
MPN16	0	13.7	0	9.59	0.00	0.00	11.65	2.91
MPN17	4.76	3.17	4.76	4.76	4.76	0.00	3.97	1.12
MPN18	3.17	4.76	4.76	9.52	3.97	1.12	7.14	3.37
				**Mean**	3.51		8.99	
				**SD**	2.50		3.38	

Supplementary Table 3: False positive error rates (FPR) for each SNV assay were determined in K562 single-cells in a patient-specific multiplex experiment.

Patient	Probe	# tested cells	FPR	%FPR	Mean %FPR	SD %FPR
MPN01	*CNOT2*	77	0	0.000	0.487	0.894
	CUL9	88	0	0.000		
	SF3B1	88	1	1.136		
	ITK	88	0	0.000		
	LPO	88	2	2.273		
	ARMCX5	88	0	0.000		
	TET2_4	88	0	0.000		
MPN04	*TRPM5*	85	5	5.882	2.647	3.094
	SUZ12	85	4	4.706		
	FGF1	85	0	0.000		
	NRAS	85	0	0.000		
MPN05	*GALNT6*	88	0	0.000	1.136	1.968
	SORCS	88	3	3.409		
	ALDH12	88	0	0.000		
MPN10	*LRCC32*	88	4	4.545	1.515	1.990
	NECAB3	88	1	1.136		
	ZMYND15	88	3	3.409		
	ACTL8	88	0	0.000		
	CBL	88	0	0.000		
	ALSCR11	88	0	0.000		
MPN11	*SF3B1*	48	0	0.000	0.490	0.980
	PCOLCE2	48	0	0.000		
	CHL1	48	0	0.000		
	JAK2	51	1	1.961		
MPN16	*CCDC158*	53	0	0.000	0.000	0.000
	SPARCL1	53	0	0.000		
	NPLOC4	53	0	0.000		
	MYO5B	53	0	0.000		
MPN17	*SERPINA*	87	0	0.000	0.287	0.575
	PNMA5	87	0	0.000		
	ARID2	87	1	1.149		
	ALOX12	87	0	0.000		
MPN18	*KRAS*	88	0	0.000	0.909	0.951
	JAK2_2	88	2	2.273		
	IDH2	88	0	0.000		
	PRTF1	88	1	1.136		
	PADI3	88	1	1.136		

Supplementary Table 4: Summary of quality control assessment of single-cell genotyping by multiplex qPCR. The total number of sorted control and target cells and their respective breakdown are depicted per patient and per experiment.

	**MPN01 t1**	**MPN01 t2**	**MPN04 t1**	**MPN04 t2**	**MPN05**	**MPN10 t1**	**MPN10 t2**	**MPN11 t1**	**MPN11 t2**	**MPN16**	**MPN17**	**MPN18**	**Total**
Total number of target cells	480	480	480	480	384	480	480	192	480	480	384	384	5184
Number of wells with no cells or more than 1 cell or bubbles	89	131	39	47	33	51	25	18	106	71	33	33	56.3
Number of cells constituting minor sub-clones below error rates	22	24	80	28	6	68	50	22	28	33	46	38	
Successful data collected from target cells	391	349	441	433	334	429	455	173	374	409	351	351	
Successful data collected from experiments	**369**	**325**	**420**	**414**	**328**	**361**	**405**	**151**	**346**	**376**	**305**	**313**	4113
Percent of data removed because of failure	18.5	27.3	8.1	9.8	8.6	10.6	5.2	9.4	22.1	14.8	8.6	8.6	
Percent of data removed as part of sub-clonal populations below error rates	4.6	5.0	16.7	5.8	1.6	14.2	10.4	11.5	5.8	6.9	12.0	9.9	
Percent of successful data collected from experiments	76.9	67.7	75.2	84.4	89.8	75.2	84.4	79.2	72.1	78.3	79.4	81.5	78.7

Supplementary Figures

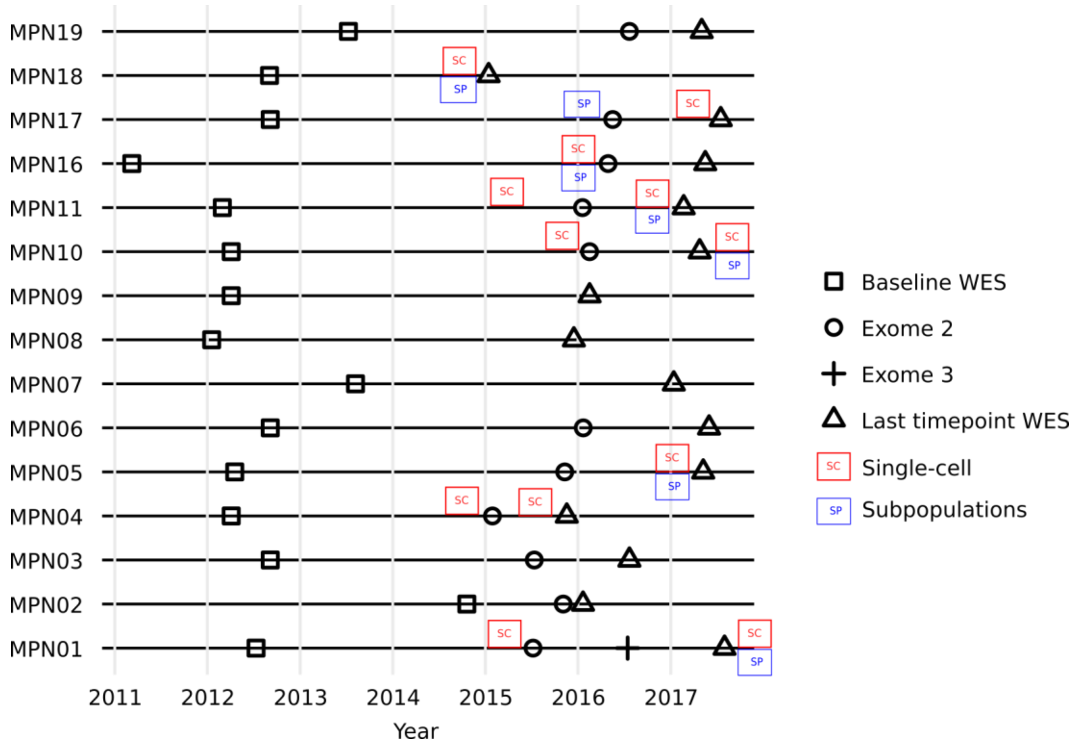

Supplementary Figure 1: CONSORT diagram depicting time points of various investigations including whole-exome sequencing (WES), single-cell genotyping (SC), and allele burden quantification in flow-sorted cell fractions (subpopulations).

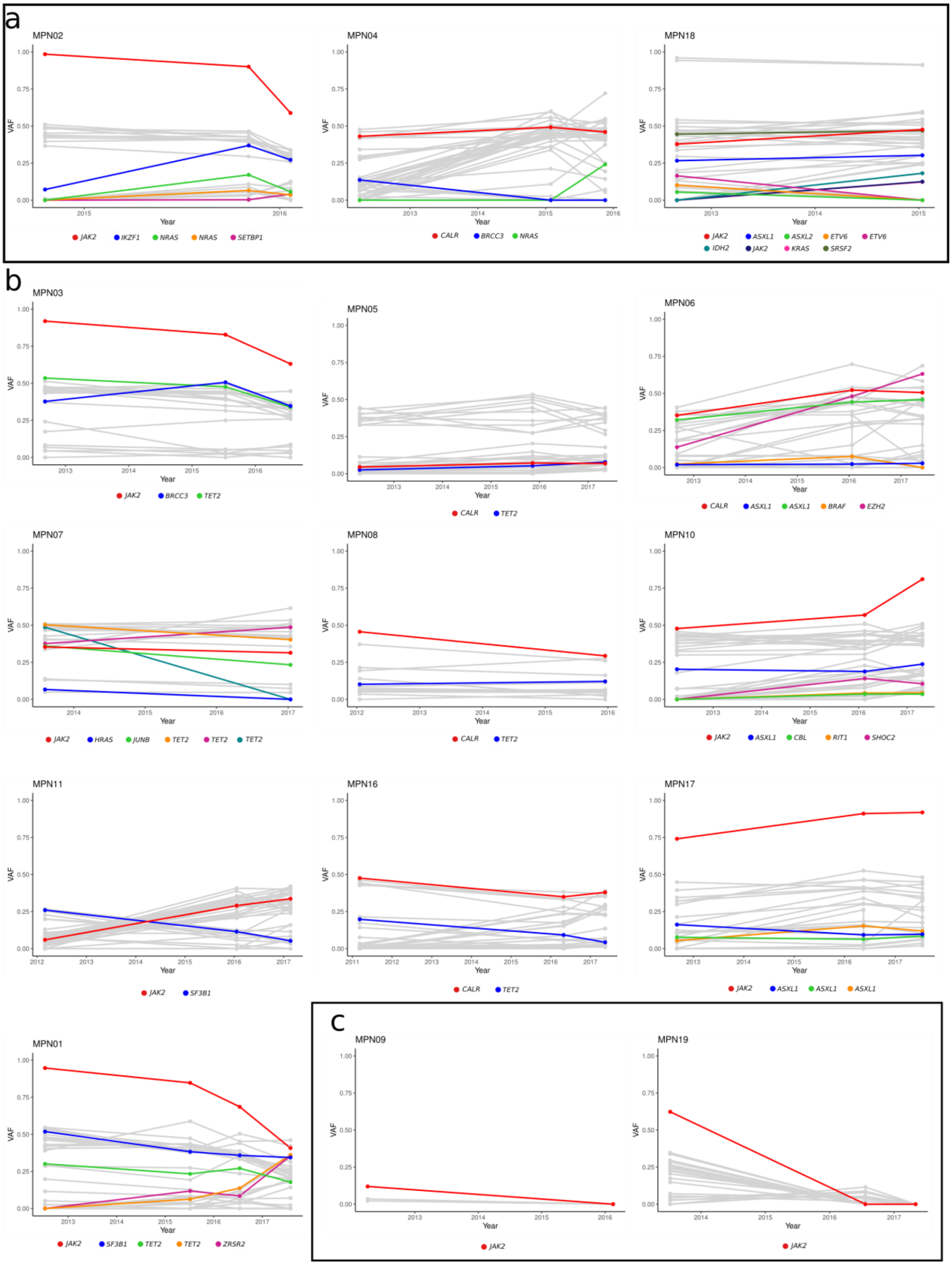

Supplementary Figure 2: Clonal dynamics in 15 MF patients based on serial WES under ruxolitinib treatment. Known cancer genes are depicted in color codes. a) Patients with AML transformation (MPN02, MPN04) or accelerated disease (MPN18). b) Clinically stable patients. c) Patients achieving molecular remission.

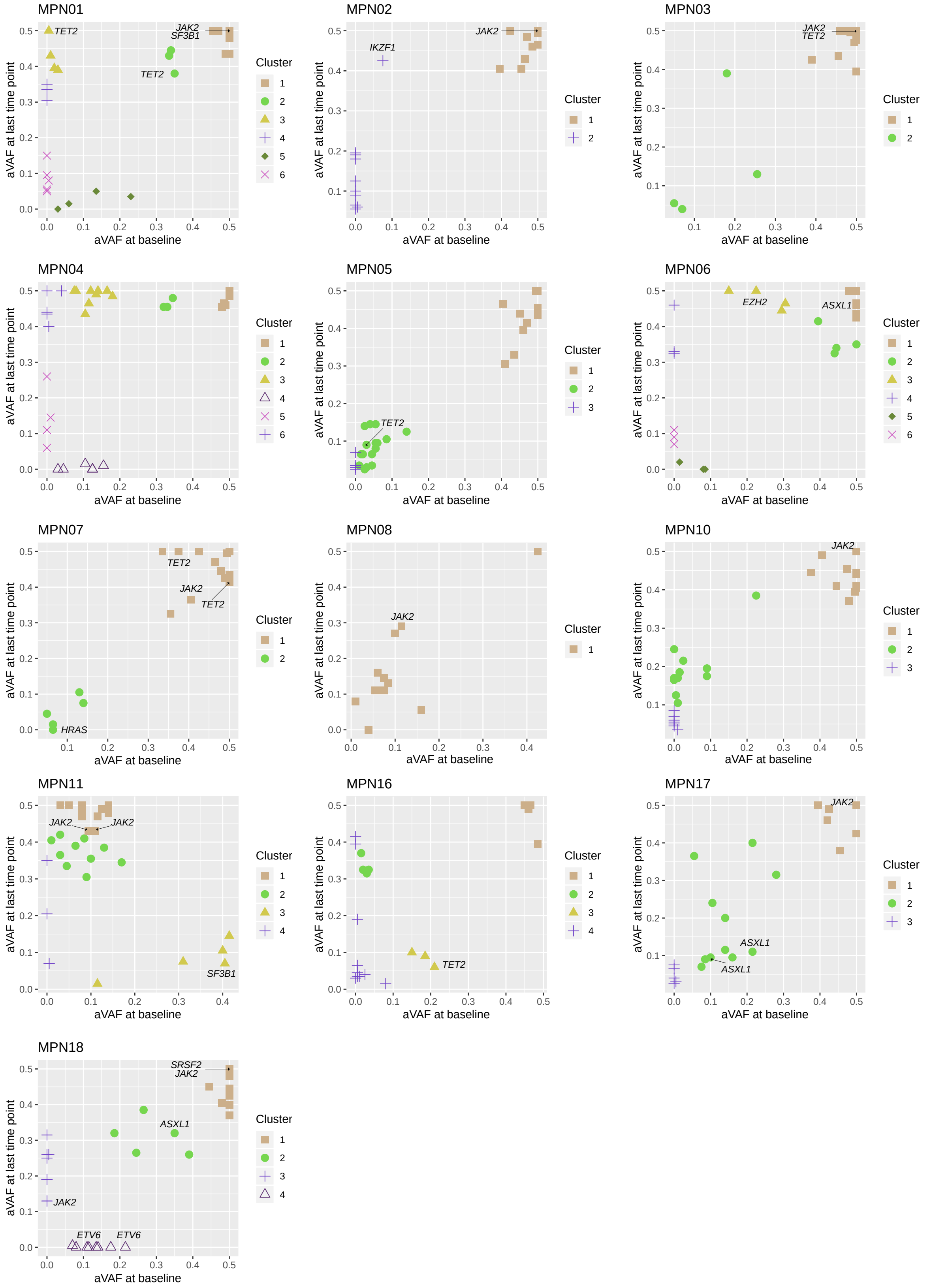

Supplementary Figure 3: Evolution of coding mutations (synonymous and non-synonymous SNVs) based on clone clustering using copy number adjusted variant allele frequencies (aVAFs). Analysis was performed on baseline and last time point WES samples using sciClone. Long insertions/deletions such as *CALR* mutations were excluded due to difficulties in accurate VAF calculation. Patients MPN09 and MPN19 were excluded due to low cancer cell fractions.

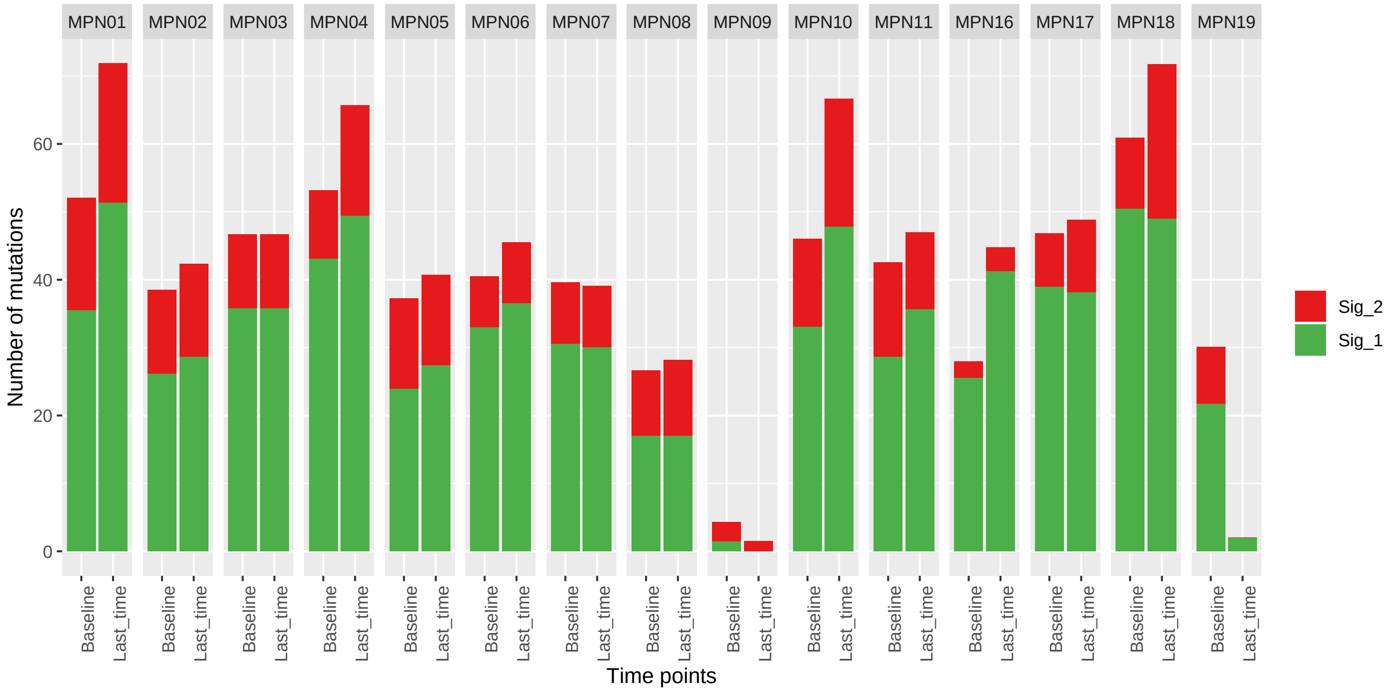

Supplementary Figure 4: Mutation signatures were analyzed using pmsignature for mutations identified in baseline and last time point WES. Somatic synonymous, nonsynonymous, and intronic variants were considered for this analysis.

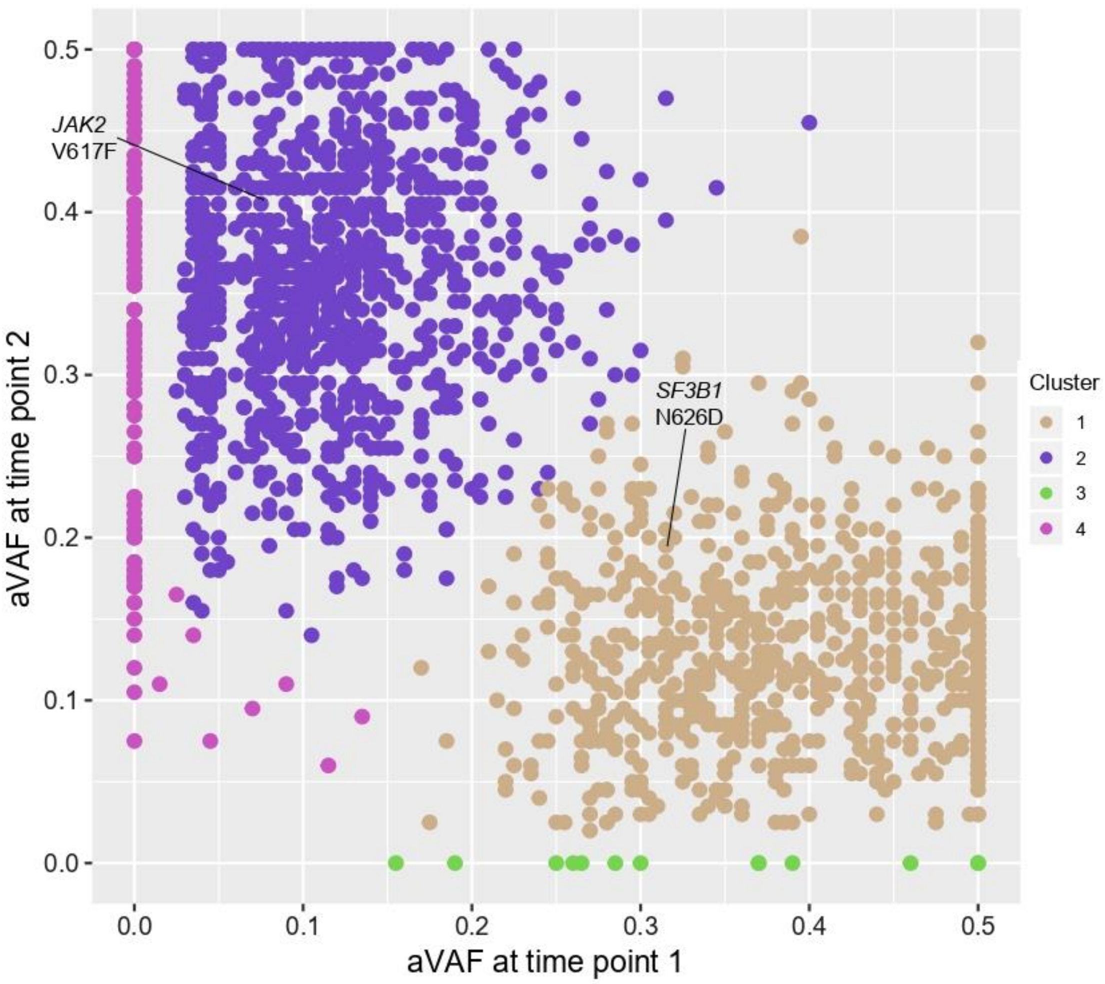

Supplementary Figure 5: Clustering according to variant allele frequencies (VAF) of acquired somatic mutations, identified by whole-genome sequencing at baseline (x-axis) and last follow-up (y-axis) time points of MPN11. These data suggest independence of respective *JAK2* V617F and *SF3B1* N626D clones

MPN01

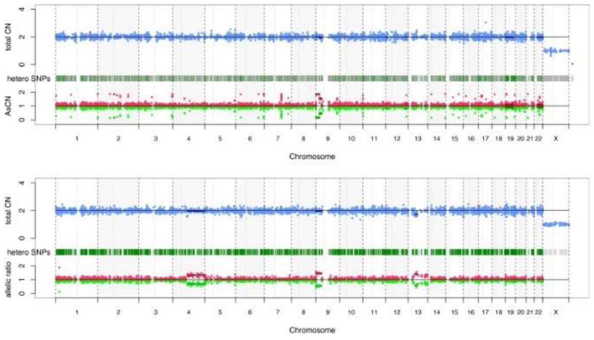

MPN02

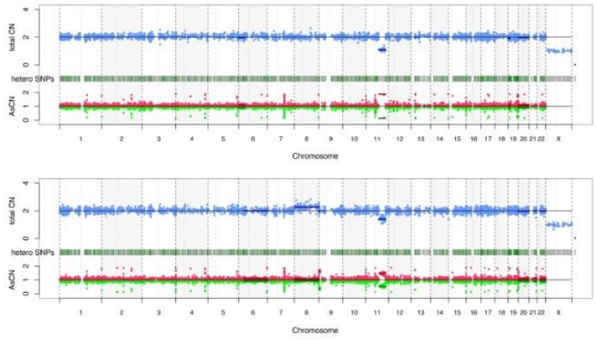

MPN03

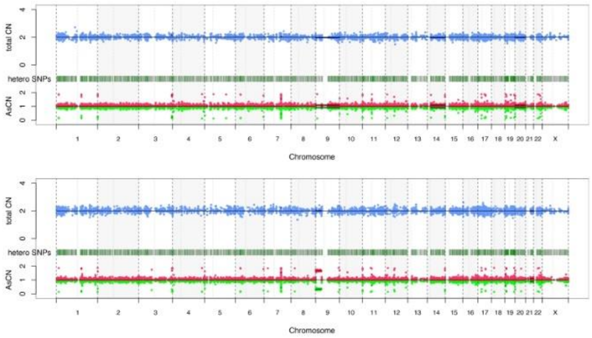

MPN04

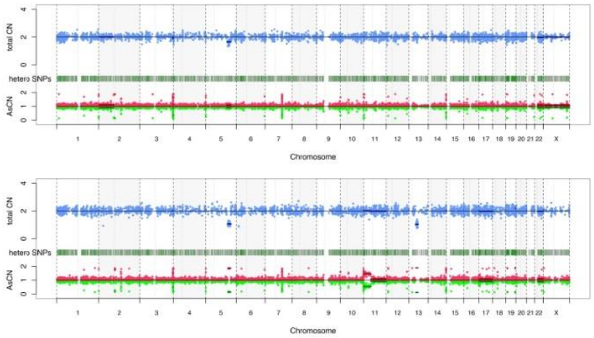

MPN05

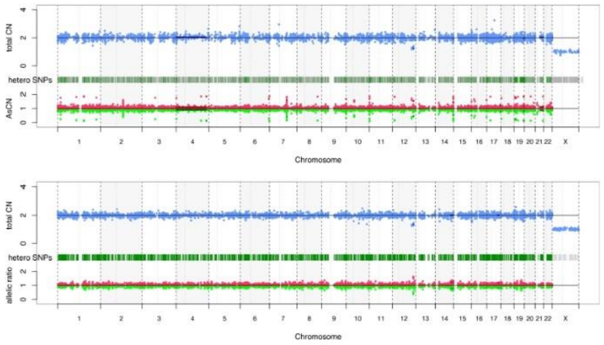

MPN06

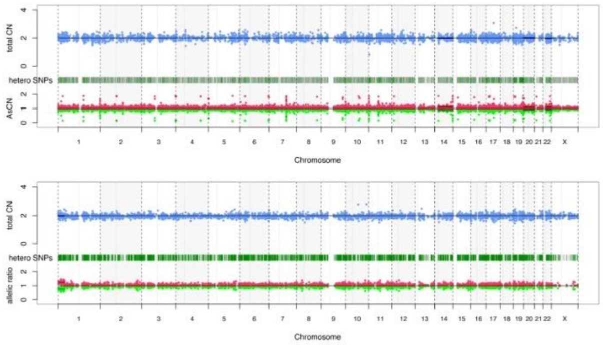

MPN07

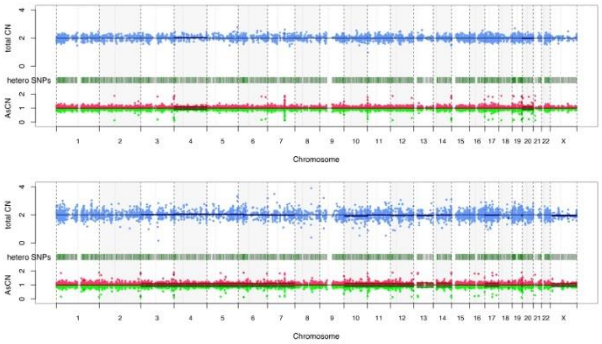

MPN08

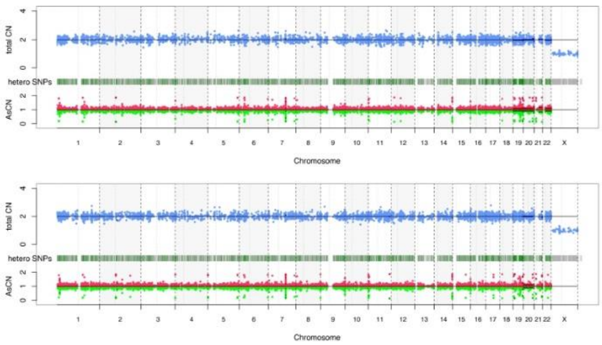

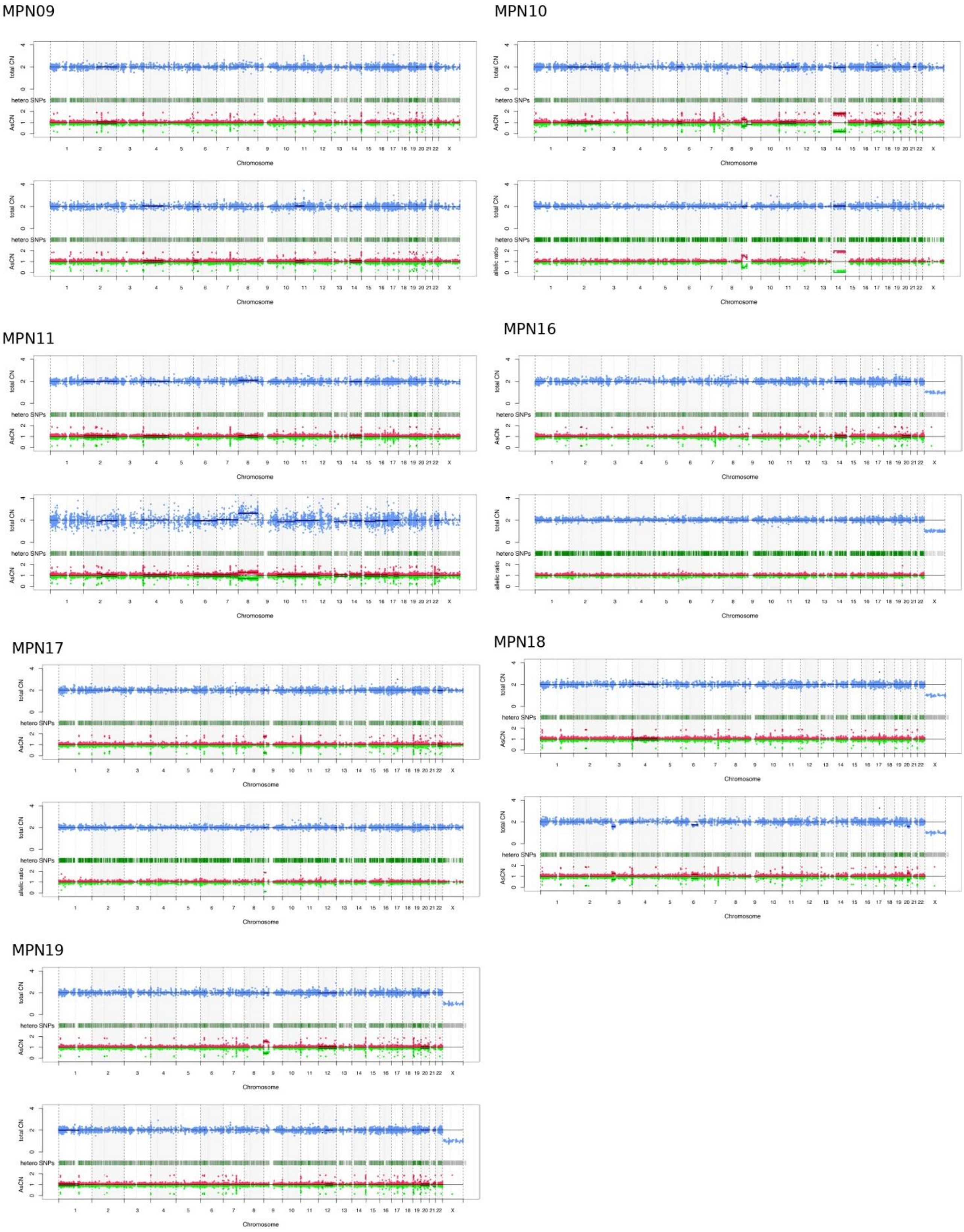

Supplementary Figure 6: Copy Number Alterations (CNA) detected by WES at baseline (upper lane) and last time point follow-up (lower lane) from 15 investigated MF patients.

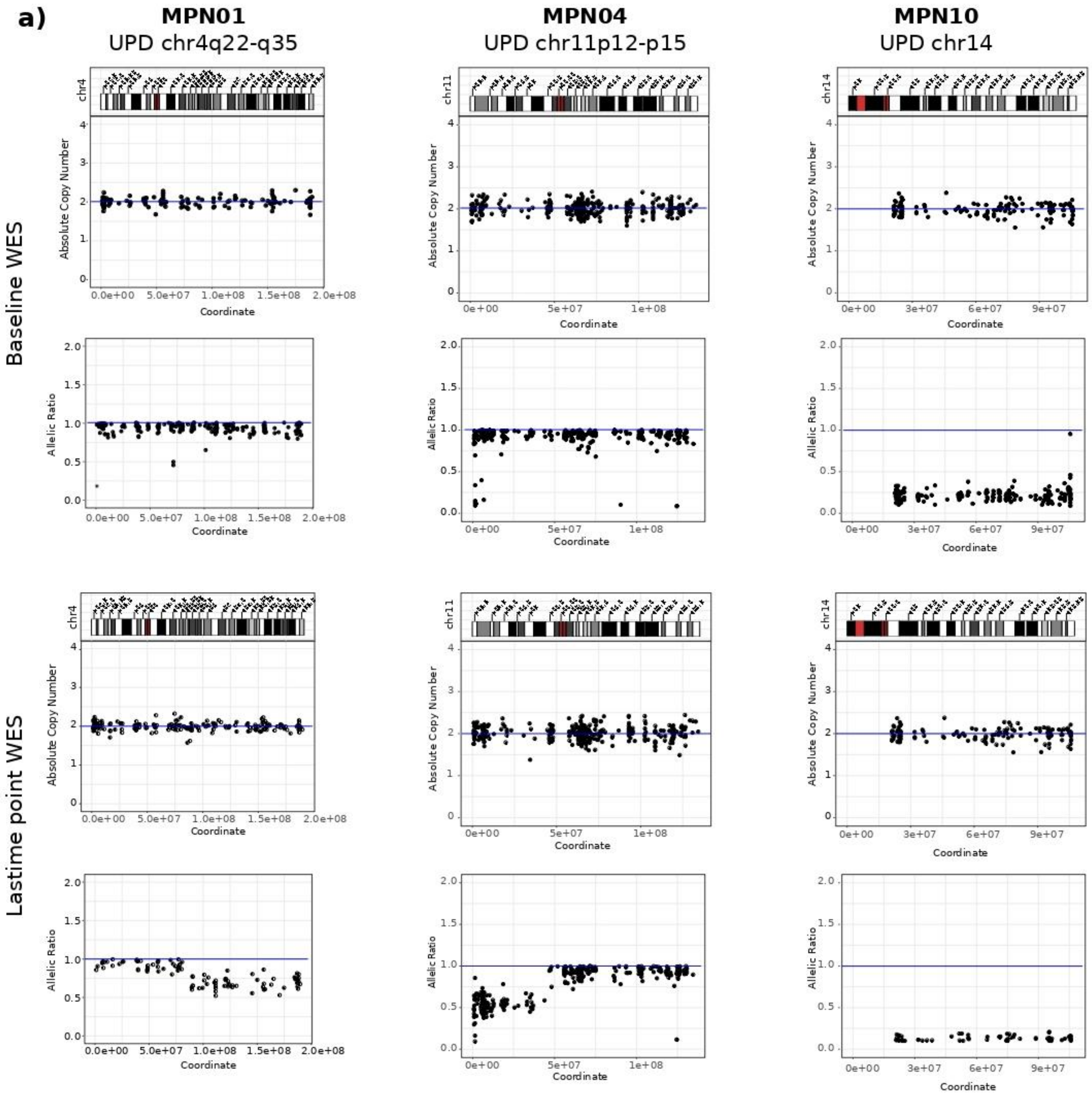
a)
MPN01
UPD chr4q22-q35
MPN04
UPD chr11p12-p15
MPN10
UPD chr14
Baseline WES
Lastime point WES
Absolute Copy Number
Allelic Ratio
Coordinate

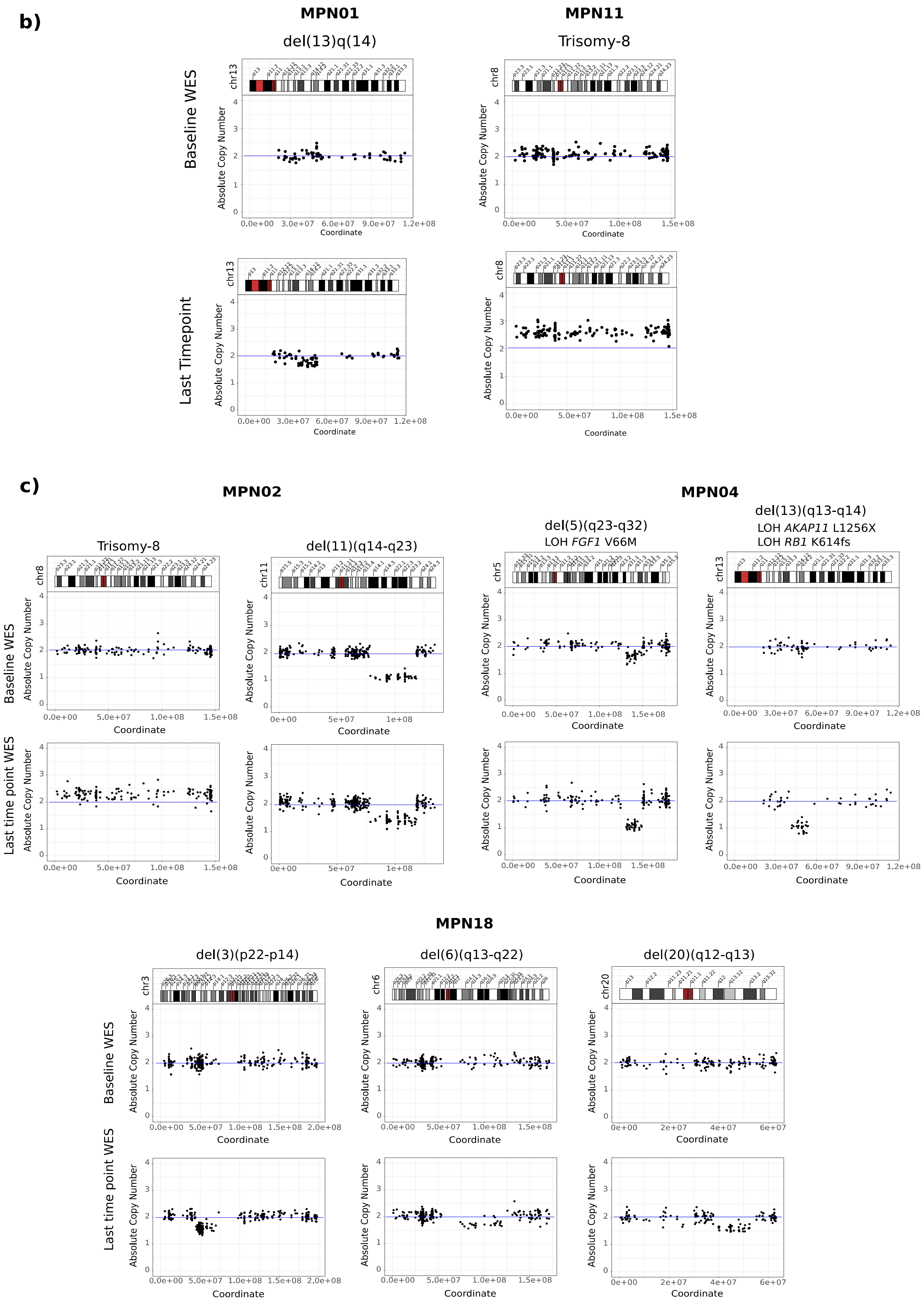

Supplementary Figure 7: a) CN-LOH/ UPD other than 9pUPD detected by WES at baseline (top) and last time point follow-up (bottom). b) Acquired CNAs found in 2 MF patients without evidence of transformation to AML/accelerated disease phase detected by WES at baseline (top) and last time point follow-up (bottom). c) CNAs found in 3 MF patients that transformed to AML/accelerated disease phase detected by WES at baseline (top) and last time point follow-up (bottom).

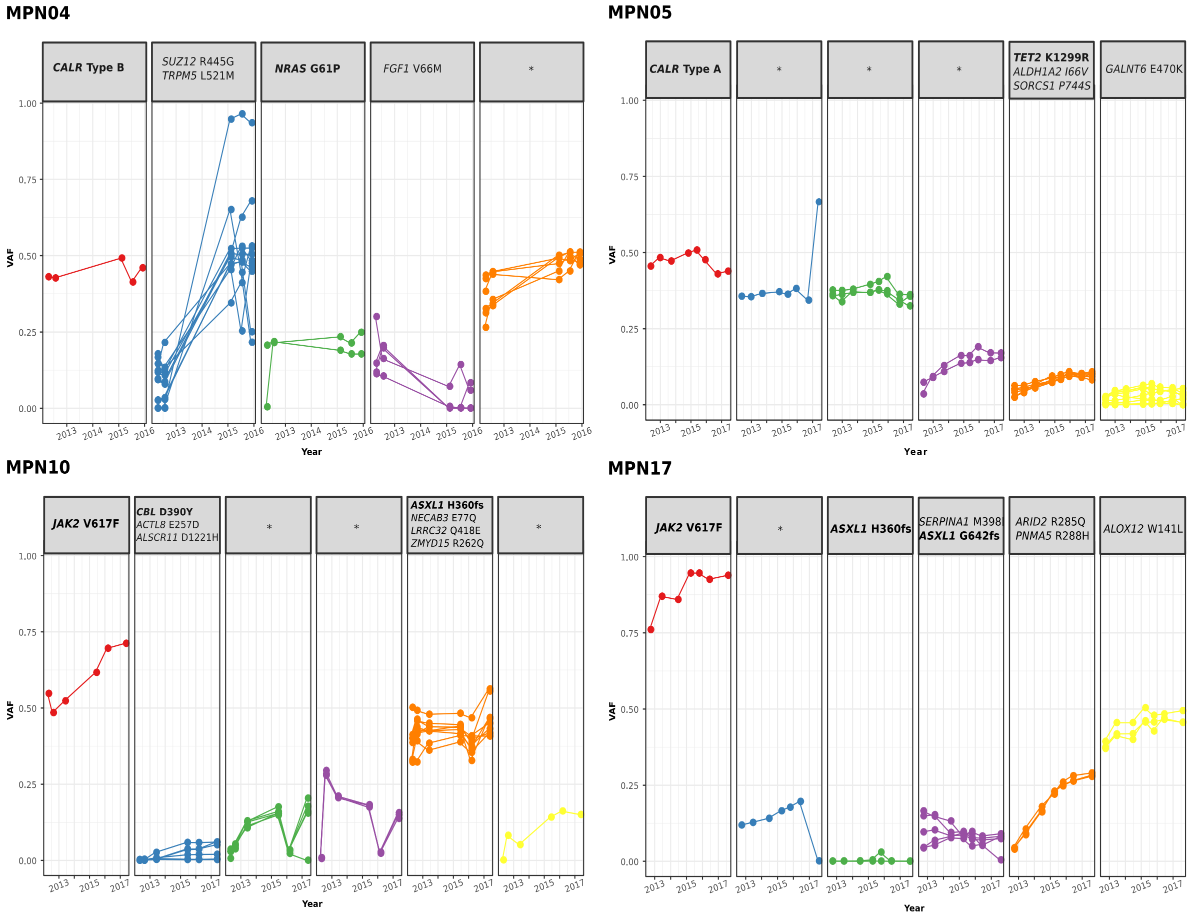

Supplementary Figure 8: VAF based clonal evolution analysis from ultra-deep sequencing at various follow-up time points. From each cluster representative mutated genes were selected. Disease-defining mutations in JAK2/*CALR* are depicted independently to emphasize their specific role in disease pathogenesis. Inference of clonal composition and evolution was performed with Sciclone (https://github.com/genome/sciclone) and ClonEvol packages (https://github.com/hdng/clonevol). Clones were manually inspected and adjusted

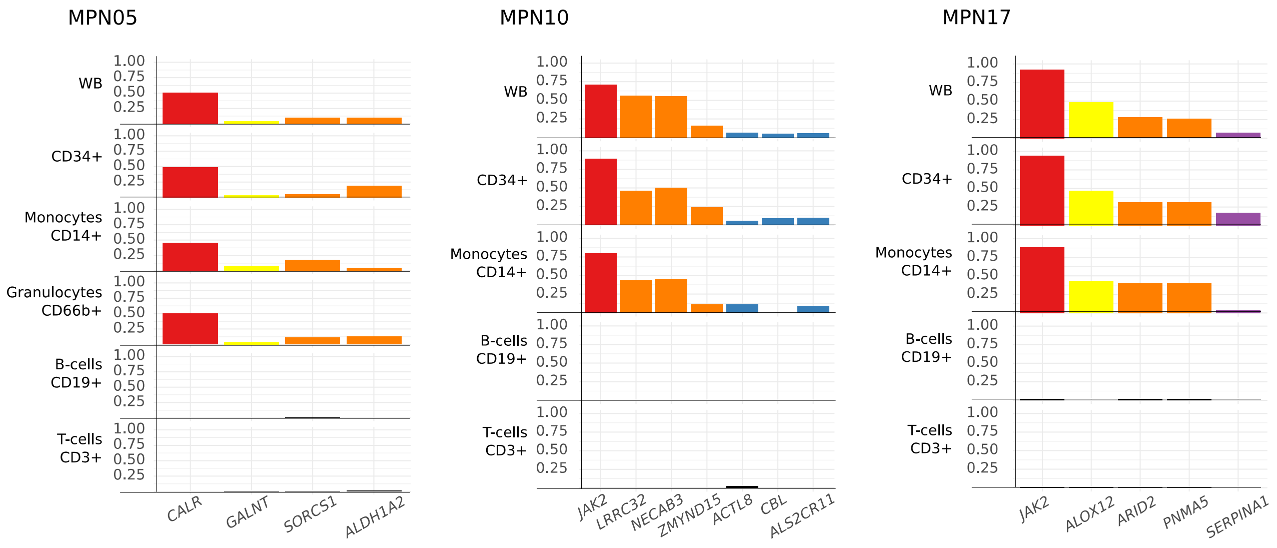

Supplementary Figure 9: Mutation quantification in flow-sorted cell fractions. Color codes correspond to respective clones shown in Supplementary Figure 8

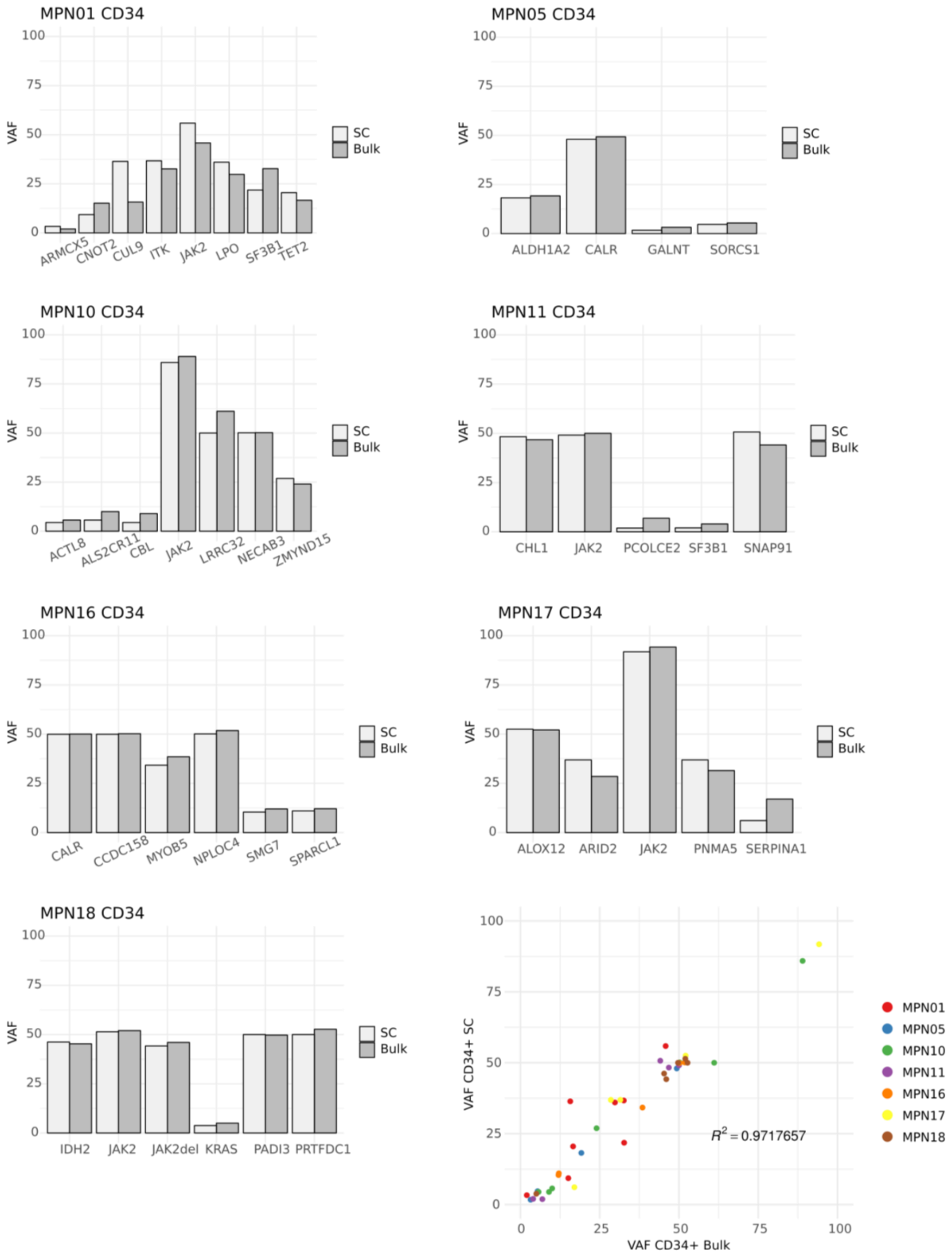

Supplementary Figure 10: Correlation allele burdens generated by ultra-deep sequencing of bulk and single-cell genotyping of flow-sorted CD34+ progenitors per patient. These data reveal a high concordance between both methods (r^2= 0.97).

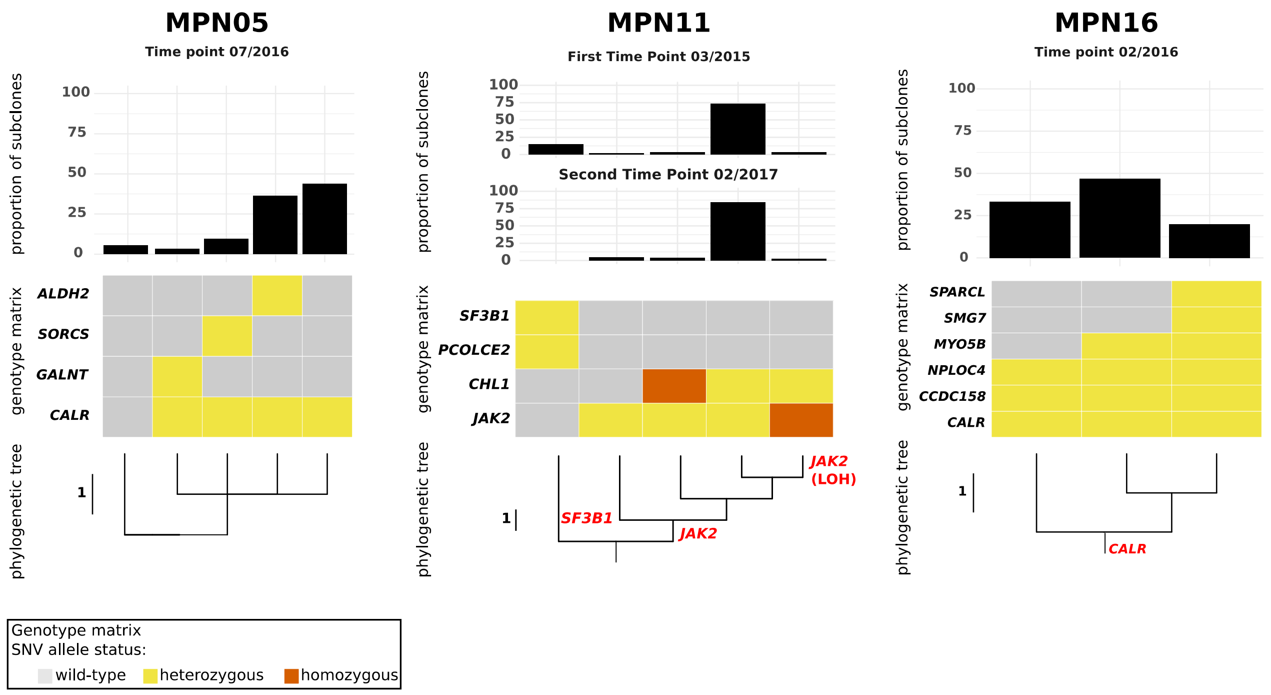

Supplementary Figure 11: Phylogenetic Trees of CD34+ progenitors in MF and proportion of clones.

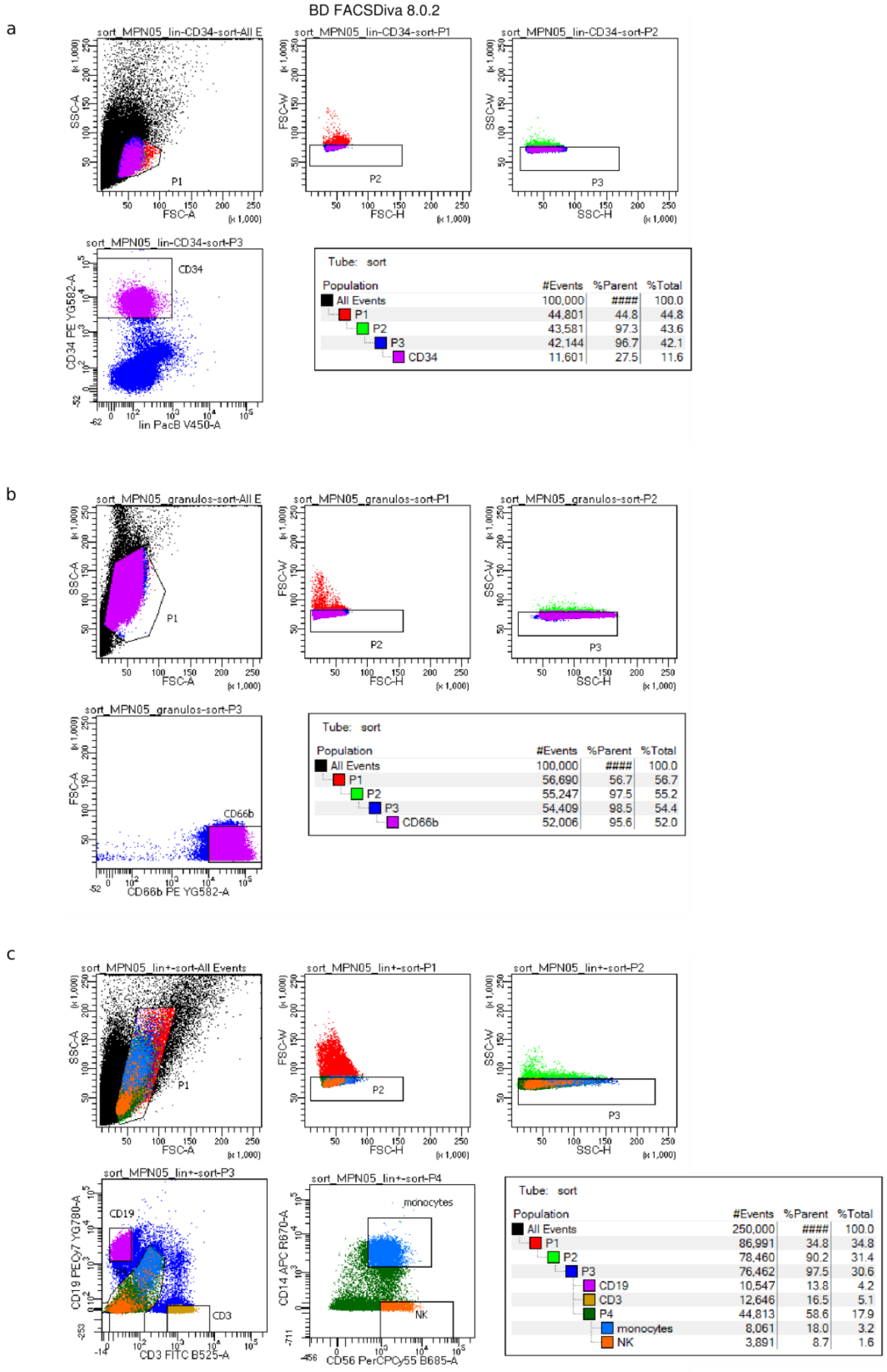

Supplementary Figure 12: Gating strategy for multicolor flow cytometry and subsequent flow-sorting. a) strategy for lineage negative CD34+ cells. b) strategy for granulocytes (CD66b). c) strategy for B-cells (CD19), T-cells(CD3), monocytes (CD14), NK-cells (CD56). CD56 fraction was not included in subsequent experiments due to lack of successful gating in most samples.

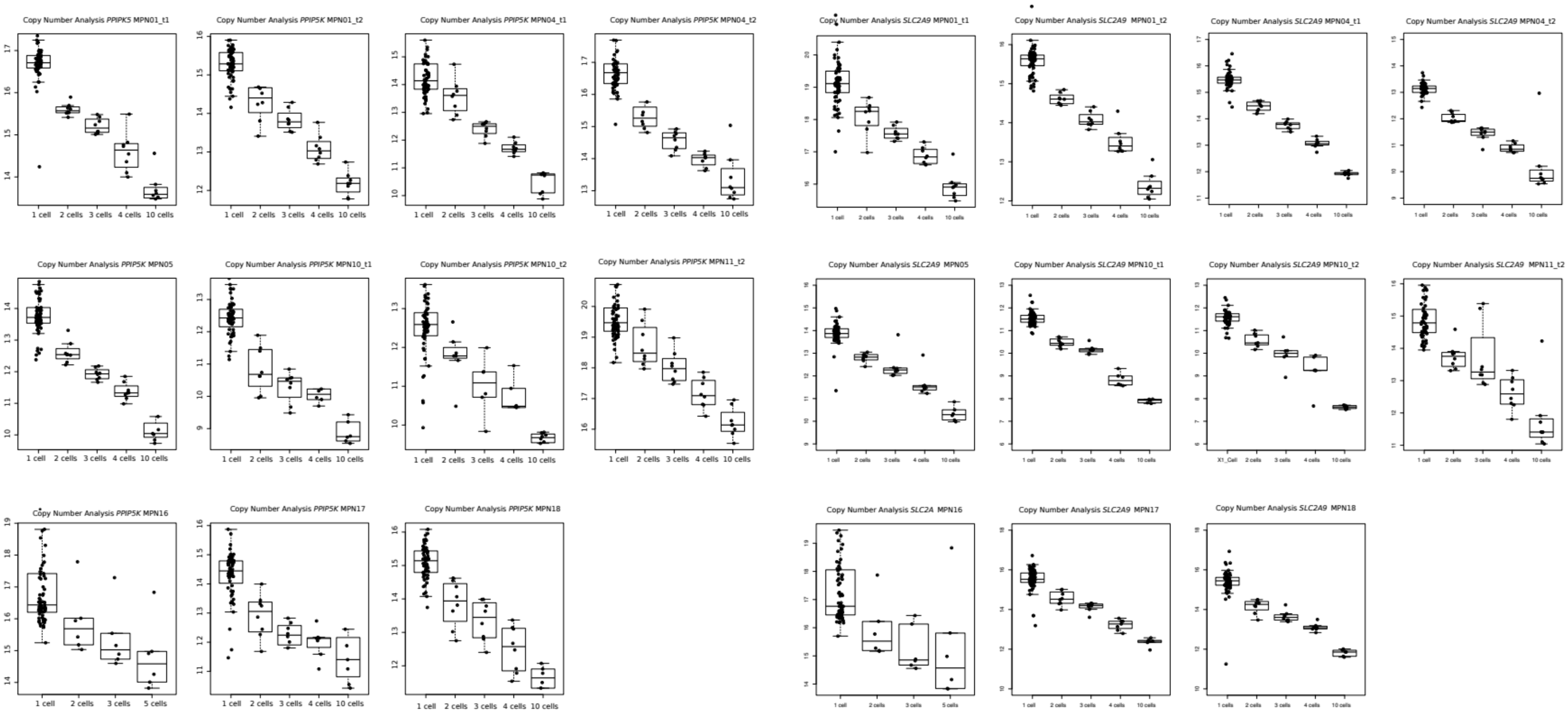

Supplementary Figure 13: Quality control of single-cell sorting for all specimens from 8 MF patients. For each patient a calibration plate was sorted including wells with 1, 2, 3, 4, and 5 or 10 cells. Two TaqMan copy number probes for (a)PPIPKa and (b)SLC2A9 were used.

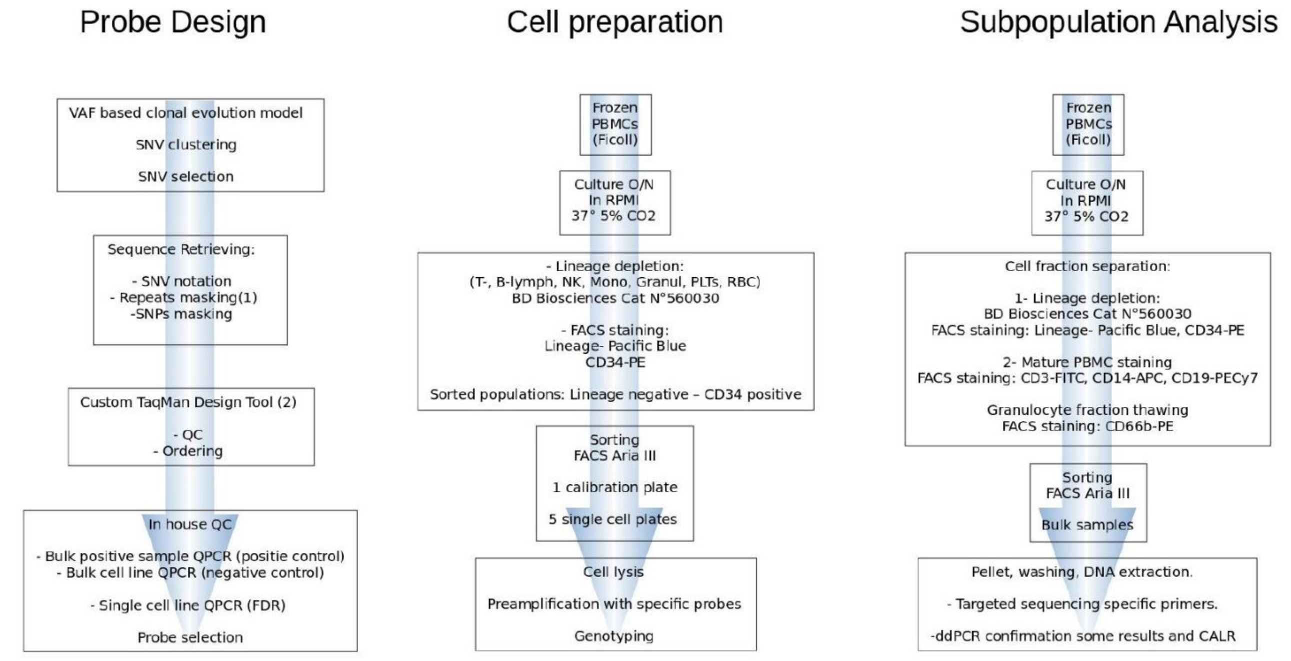

1) http://www.repeatmasker.org/cgi-bin/WEBRepeatMasker
2) http://www.thermofisher.com/taqmansnpdesign

Supplementary Figure 14: Experimental work flow of single-cell and subpopulation experiments. Three main steps are involved in theses analyses. Probe design and mutation selection, single-cell preparation and allele burden quantification in flow-sorted cell fractions
