# Supplementary material for: Single-cell analysis based dissection of clonality in myelofibrosis
Source: Nat Commun. 2020 Jan 7;11:73. doi: 10.1038/s41467-019-13892-x (PMC6946829; doi:10.1038/s41467-019-13892-x)
Supplement: Supplementary file 3 — Description of Additional Supplementary Files [file 41467_2019_13892_MOESM3_ESM.pdf]

Description of Additional Supplementary Files

File Name: Supplementary Data 1
Description: Sequencing quality criteria for whole exome sequencing and variants detected by whole exome sequencing in 15 myelofibrosis at baseline and during follow-up.

File Name: Supplementary Data 2
Description: Primers indicating respective sequences used for targeted sequencing.

File Name: Supplementary Data 3
Description: Custom TaqMan probes indicating respective sequences and commercially available probes used for single-cell analysis.
