# Supplementary material for: Single-cell analysis based dissection of clonality in myelofibrosis
Source: Nat Commun. 2020 Jan 7;11:73. doi: 10.1038/s41467-019-13892-x (PMC6946829; doi:10.1038/s41467-019-13892-x)
Supplement: Supplementary file 7 — Reporting Summary [file 41467_2019_13892_MOESM7_ESM.pdf]

nature research

Corresponding author(s): Frederik DAMM, NCOMMS-19-20549

Last updated by author(s): Nov 13, 2019

Reporting Summary

Nature Research wishes to improve the reproducibility of the work that we publish. This form provides structure for consistency and transparency in reporting. For further information on Nature Research policies, see Authors & Referees and the Editorial Policy Checklist.

Statistics

For all statistical analyses, confirm that the following items are present in the figure legend, table legend, main text, or Methods section.

n/a	Confirmed	
☒	☐	The exact sample size (n) for each experimental group/condition, given as a discrete number and unit of measurement
☒	☐	A statement on whether measurements were taken from distinct samples or whether the same sample was measured repeatedly
☒	☐	The statistical test(s) used AND whether they are one- or two-sided<br>*Only common tests should be described solely by name; describe more complex techniques in the Methods section.*
☒	☐	A description of all covariates tested
☒	☐	A description of any assumptions or corrections, such as tests of normality and adjustment for multiple comparisons
☒	☐	A full description of the statistical parameters including central tendency (e.g. means) or other basic estimates (e.g. regression coefficient) AND variation (e.g. standard deviation) or associated estimates of uncertainty (e.g. confidence intervals)
☒	☐	For null hypothesis testing, the test statistic (e.g. F, t, r) with confidence intervals, effect sizes, degrees of freedom and P value noted<br>*Give P values as exact values whenever suitable.*
☒	☐	For Bayesian analysis, information on the choice of priors and Markov chain Monte Carlo settings
☒	☐	For hierarchical and complex designs, identification of the appropriate level for tests and full reporting of outcomes
☒	☐	Estimates of effect sizes (e.g. Cohen's d, Pearson's r), indicating how they were calculated

Our web collection on statistics for biologists contains articles on many of the points above.

Software and code

Policy information about availability of computer code

Data collection	No software used
Data analysis	Detailed descriptions of the analysis have been provided in Online Methods. Sequencing data was processed using our in-house pipeline Genomon2. Copy number analysis using sequencing data was performed by in-house pipeline CNACS. Mutational signature was evaluated using pmsignature.<br><br>List of programs and softwares used for SNV calling/exome analysis:<br>Genomon2: version 2 (https://genomon.readthedocs.io/ja/latest)<br>GenomonMutationFilter: (https://github.com/Genomon-Project/GenomonMutationFilter)<br>Burrows-Wheeler Aligner: version 0.7.10 (https://sourceforge.net/projects/bio-bwa/)<br>picard-tools: version 1.39 (http://picard.sourceforge.net/)<br>EBCall: version 2 (https://github.com/friend1ws/EBCall)<br>CNACS: (https://github.com/papaemmelab/toil_cnacs)<br>pmsignature: version 0.3.0 (https://github.com/friend1ws/pmsignature)<br><br>R Tools (Analysis and Visualization):<br>MAFTools v.1.9.20 2015-12-14 https://github.com/PoisonAlien/maftools<br>GenVisR v.1.8.1 https://github.com/griffithlab/GenVisR<br>clonevol v.0.99.11 https://github.com/hdng/clonevol<br>sciClone v.1.1.0 https://github.com/genome/sciclone<br>ggtree v.1.10.5 https://guangchuangyu.github.io/ggtree<br>ggplot2 v.3.1.1 https://github.com/tidyverse/ggplot2<br>Phylognetic Analysis:<br>SiFit Genome Biology 2017,18:178 https://github.com/KChen-lab/SiFit<br>Single Cell Analysis and QPCR analysis:

Biomark & EP1 Software v4.5.2 https://www.fluidigm.com/software
StepOne(TM)Software v.2.3 Life Technologies
QuantaSoft(TM) v.1.7.4.0917 BioRad

For manuscripts utilizing custom algorithms or software that are central to the research but not yet described in published literature, software must be made available to editors/reviewers. We strongly encourage code deposition in a community repository (e.g. GitHub). See the Nature Research guidelines for submitting code & software for further information.

Data

Policy information about availability of data

All manuscripts must include a data availability statement. This statement should provide the following information, where applicable:

- Accession codes, unique identifiers, or web links for publicly available datasets
- A list of figures that have associated raw data
- A description of any restrictions on data availability

TO IMPROVE: The authors declare that the data supporting the findings of this study are available within the paper and its extended data files. All baseline and last time point WES data have been uploaded on EGA (Accession ID: EGAS00001003829). Source data underlying Figs. 1-5 and Supplementary Figs. 1-11 and 13 are provided as a Source Data file.

Field-specific reporting

Please select the one below that is the best fit for your research. If you are not sure, read the appropriate sections before making your selection.

☒ Life sciences ☐ Behavioural & social sciences ☐ Ecological, evolutionary & environmental sciences

For a reference copy of the document with all sections, see nature.com/documents/nr-reporting-summary-flat.pdf

Life sciences study design

All studies must disclose on these points even when the disclosure is negative.

Sample size	A total of 15 patients with myelofibrosis were included. For such a rare disease, it is an important cohort size.
Data exclusions	No data were excluded
Replication	No replications of sequencing experiments werde undertaken. However, some samples were analyzed by two different sequencing techniques and these results were checked for correlation. In all of these experiemtns, a very high correlation was observed (e.g. Supplementary Figure 10)
Randomization	No randomization or allocation was undertaken
Blinding	As no allocation has been undertaken, no blinding was necessary

Reporting for specific materials, systems and methods

We require information from authors about some types of materials, experimental systems and methods used in many studies. Here, indicate whether each material, system or method listed is relevant to your study. If you are not sure if a list item applies to your research, read the appropriate section before selecting a response.

Materials & experimental systems

n/a	Involved in the study	
☐	☒	Antibodies
☒	☐	Eukaryotic cell lines
☒	☐	Palaeontology
☒	☐	Animals and other organisms
☐	☒	Human research participants
☒	☐	Clinical data

Methods

n/a	Involved in the study	
☒	☐	ChIP-seq
☐	☒	Flow cytometry
☒	☐	MRI-based neuroimaging

Antibodies

Antibodies used	CD34-PE (dil 1:5) BD catalog: 555822 host: Mouse, clone: 581<br>Streptavidine-BrilliantViolet (dil 1:500) Biolegend catalog: 405225<br>CD66b-PE (dil 1:60) BD catalog: 561650, clone: G10F5<br>CD3-FITC (dil 1:40) BD catalog: 555339, clone: HIT3a<br>CD19-PECy7 (dil 1:100) BD catalog: 560728, clone: HIB19

	CD14-APC (dil 1:20) eBioscience catalog: 17-0149, clone: 61D3
Validation	Commercially validated antibodies, all antibodies were tested "in house" for determination of dilutions, buffers and sorter compatibility. Single and multiplexed stainings were tested.

Human research participants

Policy information about studies involving human research participants

Population characteristics	15 MF patients (PMF n=8; post-ET/PV-MF n=7; median age 66 years) were included at initiation of ruxolitinib treatment. PB was sampled serially in a prospective manner (mean follow-up time: 3.9 years/patient)
Recruitment	Only patient recruitment criteria was written informed consent and treatment with the JAK inhibitor ruxolitinib.
Ethics oversight	The study was conducted in accordance with the Declaration of Helsinki and with ethical approval obtained from the local ethics committee of the Charité - Universitätsmedizin Berlin in Germany. All patients provided written informed consent.

Note that full information on the approval of the study protocol must also be provided in the manuscript.

Flow Cytometry

Plots

Confirm that:

☒ The axis labels state the marker and fluorochrome used (e.g. CD4-FITC).

☒ The axis scales are clearly visible. Include numbers along axes only for bottom left plot of group (a 'group' is an analysis of identical markers).

☐ All plots are contour plots with outliers or pseudocolor plots.

☒ A numerical value for number of cells or percentage (with statistics) is provided.

Methodology

Sample preparation	Lineage negative(Lin-) CD34+ cells: PBMCs cells were thawed at 37° C and cultured over-night in RPMI medium, supplemented with 10% FBS and 1X Streptavidin and 1X Penicilin/Streptomycin at 5% CO2, 37°C. Cells were washed and lineage positive cells were labeled using Human Cell Depletion Set (BD Biosciences). Lin + cells were depleted using streptavidin coated magnetic beads. The supernatant containing Lin - cells was washed and labeled with anti-human CD34-PE antibodies (BD Biosciences). Remaining Lin + cells were labeled with Streptavidin-BrilliantViolet (BD Biosciences) conjugate for cytometric exclusion. Lin - / CD34 + single-cells were sorted in 96 well plates containing 2.5µL of lysis buffer. All the procedures were performed at 4° C until completion of cell lysis.<br><br>Mature blood cell lineages: PBMCs were thawed and cultured overnight at standard conditions. Cells were then separated using the first fraction for Lin - /CD34 + sorting and the second fraction for sorting mature cell populations. For the later, cells were labeled with the following conjugated anti-human antibodies: FITC-CD3, APC-CD14, PE/Cy7-CD19 (BD Biosciences). If available, previously Ficoll-enriched granulocytes were thawed and labeled with anti-human CD66b-PE (BD Biosciences).
Instrument	FACSAriaII SORP
Software	FACSDiva 8.0.2
Cell population abundance	Sorted population were reanalized for purity stimation after sorting. Purity ranged from 95% to 100% for each population. Sorting reports were generated for each sample.
Gating strategy	Lineage negative (Lin-) CD34+: Living cells(P1): FSC-A/SSC-A; removal duplets-1(P2): FSC-H/FSC-W, removal duplets-2(P3) SSC-H/SSC-W; Lineage negative CD34+ progenitors: lin-PB (V450-A)/CD34-PE(YG582-A).<br>Granulocytes: Living cells(P1): FSC-A/SSC-A; removal duplets-1(P2): FSC-H/FSC-W, removal duplets-2(P3) SSC-H/SSC-W; granulocytes: CD66b-PE (YG582-A)/FSC-A.<br>PBMCs: FSC-A/SSC-A; removal duplets-1(P2): FSC-H/FSC-W, removal duplets-2(P3) SSC-H/SSC-W; B-cells (CD19+, CD3-,CD14-) CD19-PECy7(YG780-A)/ T-cells (CD3+, CD19-, CD14-), CD3-FITC(B525A)/ Monocytes (CD14+, CD3-, CD19-) CD14-APC(R670-A).

☒ Tick this box to confirm that a figure exemplifying the gating strategy is provided in the Supplementary Information.
